# Photoreceptor phagosome processing defects and disturbed autophagy in retinal pigment epithelium of *Cln3^Δex1-6^* mice modelling juvenile neuronal ceroid lipofuscinosis (Batten disease)

**DOI:** 10.1093/hmg/ddv406

**Published:** 2015-10-08

**Authors:** Silène T. Wavre-Shapton, Alessandra A. Calvi, Mark Turmaine, Miguel C. Seabra, Daniel F. Cutler, Clare E. Futter, Hannah M. Mitchison

**Affiliations:** 1 UCL Institute of Ophthalmology, University College London, London EC1V 9EL, UK,; 2 Molecular Medicine, National Heart and Lung Institute, Imperial College London, London SW7 2AZ, UK,; 3 Genetics and Genomic Medicine Programme and Birth Defects Research Centre, Institute of Child Health, University College London, London WC1N 1EH, UK,; 4 Nuclear Dynamics and Architecture, Institute of Medical Biology, Singapore 138648, Singapore,; 5 Faculty of Life Sciences, Division of Biosciences and; 6 Department of Cell and Developmental Biology, University College London, London WC1E 6BT, UK and; 7 MRC Cell Biology Unit, MRC Laboratory for Molecular Cell Biology, London, UK

## Abstract

Retinal degeneration and visual impairment are the first signs of juvenile neuronal ceroid lipofuscinosis caused by *CLN3* mutations, followed by inevitable progression to blindness. We investigated retinal degeneration in *Cln3^Δex1-6^* null mice, revealing classic ‘fingerprint’ lysosomal storage in the retinal pigment epithelium (RPE), replicating the human disease. The lysosomes contain mitochondrial F_0_-ATP synthase subunit c along with undigested membranes, indicating a reduced degradative capacity. Mature autophagosomes and basal phagolysosomes, the terminal degradative compartments of autophagy and phagocytosis, are also increased in *Cln3^Δex1^*^-*6*^ RPE, reflecting disruption to these key pathways that underpin the daily phagocytic turnover of photoreceptor outer segments (POS) required for maintenance of vision. The accumulated autophagosomes have post-lysosome fusion morphology, with undigested internal contents visible, while accumulated phagosomes are frequently docked to cathepsin D-positive lysosomes, without mixing of phagosomal and lysosomal contents. This suggests lysosome-processing defects affect both autophagy and phagocytosis, supported by evidence that phagosomes induced in *Cln3^Δex1^*^-^*^6^*-derived mouse embryonic fibroblasts have visibly disorganized membranes, unprocessed internal vesicles and membrane contents, in addition to reduced LAMP1 membrane recruitment. We propose that defective lysosomes in *Cln3^Δex1^*^-^*^6^* RPE have a reduced degradative capacity that impairs the final steps of the intimately connected autophagic and phagocytic pathways that are responsible for degradation of POS. A build-up of degradative organellar by-products and decreased recycling of cellular materials is likely to disrupt processes vital to maintenance of vision by the RPE.

## Introduction

Lysosome storage disorders (LSDs) are a group of >60 inherited metabolic and neurological conditions (1,2) that collectively form a prominent cause of childhood neurodegeneration (3,4). The genetic mutations underlying LSDs disrupt lysosome-associated catabolic pathways involved in breakdown of cellular materials, classically manifesting with lysosomal accumulation (storage) of undegraded waste products (5). In LSDs, disrupted lysosome function affects the degradation of intracellular materials by macroautophagy (hereafter referred to as autophagy) (6,7), degradation of endocytosed extracellular materials and several other lysosomally-directed biological processes including cell and plasma membrane homeostasis, nutrient sensing, energy metabolism and the immune response (8). Compromised cells show accumulations of autophagosomes, dysfunctional mitochondria and endoplasmic reticulum, ubiquitinated proteins and protein aggregates. Varied neuropathogenic consequences arise, due to metabolic insufficiency, reduced synaptic plasticity, vesicle trafficking defects, perturbed calcium signalling, increased reactive oxygen species and aberrant inflammatory and apoptotic signalling (2,5,8,9).

The neuronal ceroid lipofuscinoses (NCLs) are a prevalent class of LSD, and mutations in 13 different genes (*CLN1-CLN8* and *CLN10-CLN14*) cause a number of clinically distinct NCL subtypes. The highest incidence of disease (around 1 per 100 000 births) is reported for the three most prevalent forms caused by *CLN1*, *CLN2* and *CLN3* mutations, making them the most common neurodegenerative diseases in children (4,10,11). NCLs hallmarks are progressive cognitive deficits and visual deterioration resulting from widespread neurodegeneration, with neuronal and photoreceptor cell death in the central nervous system (CNS) and retina (11). The pathological processes underlying lysosomal storage and disease progression in the NCLs remain poorly understood, and biochemical characterization of the lysosomal storage material has not revealed the natural substrates for the enzyme-associated or other NCL subtypes. The 13 recognized NCL proteins seem to be widely expressed with diverse roles. Most but not all reside in the lysosome, and none have yet been shown to cooperate in any well-defined cellular pathways (12,13).

The commonest NCL subtype is the juvenile form caused by mutations in *CLN3*. Visual loss is almost invariably the presenting symptom of juvenile NCL, manifesting around 4–8 years of age with night blindness, photophobia and loss of peripheral and colour vision (14–17). Retinal disease progresses along a similar degenerative course to retinitis pigmentosa. There is a loss of photoreceptors and retinal pigment epithelium (RPE), culminating in widespread atrophy and severe loss of neurons from all retinal layers accompanied by severe gliosis at end stage (16,18). The decline to blindness is typically rapid (1–2 years) compared with other retinal degenerations (14,15,17), but affected individuals can often remain blind-only for a prolonged period (1–9 years) before other signs of deterioration (11,16). *CLN3* mutations in juvenile NCL cause progressive lysosomal storage of autofluorescent proteolipid lipofuscin-like pigments of a characteristic ‘fingerprint’ ultrastructure rich in subunit c of the mitochondrial F_0_-ATP (adenosine triphosphate) synthase (11). Pre-postmortem stage affected retina have only been studied in CLN3-deficient mouse models, where retinal degeneration is first indicated by widespread accumulation of this characteristic autofluorescent storage material in the photoreceptor and ganglion cell layers (16,19,20). This sequestering of an inner mitochondrial membrane protein component occurs for unknown reasons, but has long been debated to arise from deficient autophagy (21).

CLN3 is a ubiquitously expressed, highly conserved glycosylated membrane protein with cytoplasmic C- and N-terminal domains and two cytosolic lysosomal targeting motifs (22–24). It mainly localizes to late endosome and lysosome membranes, with evidence also for localization in the trans-Golgi network (TGN)/post-TGN compartments and for roles in post-Golgi trafficking (25–28). In CLN3-deficient cells there is a markedly reduced exit from the TGN of the cation-independent mannose 6-phosphate receptor that is responsible for delivery of lysosome hydrolases from the TGN to the endocytic pathway (28). CLN3-deficient cells have also been reported to display altered trafficking or activity of lysosomal enzymes (25,28–31), altered intralysosomal amino acid transport (32) and a loss of lysosome acidity coinciding with reportedly altered activity of the pH regulator vacuolar H (+)-ATPase (33–35). Correspondingly, CLN3-deficient cells display several faulty lysosome-associated processes, even prior to significant lysosome storage accumulation, notably deficient vesicle fusion and maturation events critical for autophagy (34,36), and deficient endocytosis (27,31,37,38). These suggested protein trafficking and lysosomal maintenance roles indicate involvement of CLN3 in cytoskeleton (cell morphology, migration) (27,38,39), synapse (37,40) and neurotransmission (41,42) functions. Lysosome dysfunction in some other NCL subtypes is already clearly linked to neuronal disease pathology, for example synaptic degeneration, and reduced neuronal plasticity arising from presynaptic abnormalities affecting the synaptic vesicle docking, recycling and neurotransmitter release cycle (43–45).

In the eye, the distal tips (10%) of all photoreceptor outer segments (POS) are shed on a daily basis. The phagocytosis and degradation of shed POS by the adjacent RPE cells (46,47) is essential to maintain photoreceptor excitability. Phagosomes must move into the cell body in order to undergo a ‘maturation’ process involving sequential fusions with the endocytic pathway (48,49). Fusion with lysosomes, to form phagolysosomes, delivers membrane and degradative lumenal hydrolase enzymes to the phagosomes for the breakdown and recycling of the internalized POS (49). Failure to phagocytose shed outer segments, such as in the Royal College of Surgeons rat (50,51), leads to rapid photoreceptor cell death, demonstrating the importance of clearance of spent POS by the RPE, and the essential nature of lifelong maintenance of photoreceptors for maintenance of vision. Given the huge phagocytic load of RPE cells they are likely to be particularly sensitive to defects in lysosomal delivery or lysosomal activity. However, no RPE-specific phenotype has ever been described for the NCL diseases.

An emerging field of study suggests that autophagy dysfunction can also impair RPE cell function and that deficient lysosome-autophagosome degradation could underlie a number of retinal diseases [reviewed in (52)]. Autophagy represents another branch of the lysosome-directed digestive system of cells, with autophagosomes forming to engulf and recycle cytoplasmic contents that, akin to phagocytosis, are degraded after sequential fusions with the endo-lysosomal system occur during the terminal maturation steps of the pathway (53). Autophagy in the RPE is used as in most cells to maintain cell homeostasis, and for example in age-related macular degeneration the disease-related build-up of lipofuscin in lysosomes of the RPE has been linked to abnormal autophagy. A recent study showed retinal function is decreased in autophagy-deficient mice and increased autophagy is triggered by the cyclical shedding of POS, with at least part of the autophagy machinery directly involved in phagosome maturation to affect the degradation of phagocytosed POS (54).

Since visual defects are the first manifestation of juvenile NCL, and autophagic disturbances characterize the disease neuropathology (36,55,56), we used *Cln3^Δex1^*^-^*^6^* knockout mice (57,58) to investigate whether CLN3 has a role in retinal phagosome degradation. We find deficiencies in the autophagic pathway and accumulation of characteristic (mitochondrial F_0_-ATP synthase subunit c positive) lysosomal storage material in the RPE that appears similar to that observed in the brain of this and other *Cln3*-deficient mice (57,59,60), replicating human juvenile NCL lysosomal storage and pathology (11,56). We further show that phagosome degradation is deficient, both in *Cln3^Δex1^*^-^*^6^* RPE and in a model phagocytic system derived from *Cln3^Δex1^*^-^*^6^* fibroblasts. These findings in two distinct lysosome-related degradative pathways constitute the first description of a deficit in the RPE caused by loss of CLN3. The consequences could contribute to the visual deterioration that is the first symptom of the juvenile onset subtype of NCL.

## Results

### Autophagosomes and storage material characteristic of juvenile NCL accumulate in the RPE and neurons of the brain of aged *Cln3^Δex1^*^-^*^6^* null mutants

**Figure 1.**
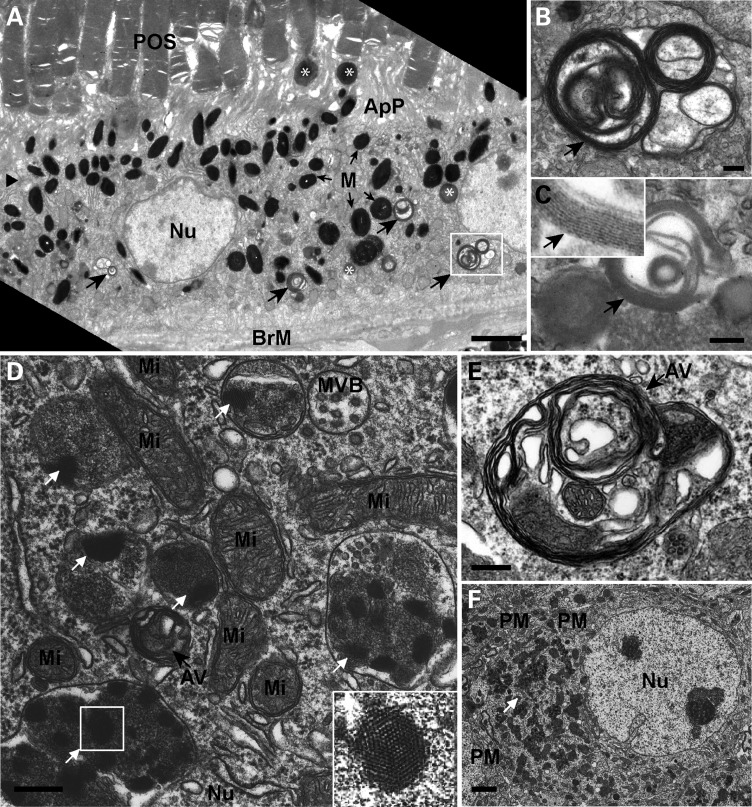
Retinal pigment epithelium and neuronal cells from brains of aged *Cln3^Δex1-6^* mice both accumulate classical fingerprint storage material and autophagsomes. Conventional EM analysis of aged *Cln3^Δex1-6^* mouse RPE (**A**–**C**) and brain cortex (**D**–**F**). (A) Lower magnification of RPE of a 12-month old *Cln3^Δex1-6^* mouse shows POS adjacent to apical processes (ApP), numerous melanosomes (M, small black arrows) and Bruch's membrane (BrM). Electron dense single-membrane phagosomes are located apically containing phagocytosed POS, and also more basally (white asterisks). Black arrowhead indicates tight junction, position of the phagosomes is recorded as apical if above the tight junction. Large black arrows indicate double membrane-bound vacuolar/autophagosome-like structures seen in the basal RPE area. Scale bar, 2 µm. (B) Enlarged box from panel A highlights an autophagosome at higher magnification, containing cytoplasm captured within multiple membranes (arrow). Scale bar, 200 nm. (C) Lipofuscin-like fingerprint structures characteristic of *Cln3*-specific disease storage material are also present in *Cln3^Δex1-6^* RPE cells (C, arrows and higher magnification inset). Scale bar, 200 nm. (D) *Cln3^Δex1-6^* cortical neuron at 12 months shows classical single membrane-bound storage material with fingerprint profiles (white arrows). A magnification of the boxed area is shown in the bottom right to highlight the fingerprint pattern of storage). Storage is found in proximity to mitochondria (Mi), and a multivesicular body (MVB), in addition to autophagosome structures (AV, black arrow). The autophagosomes are double-membrane bound and contain cytoplasm, membranes and internal vesicles. Scale bar, 200 nm. (E) A representative cortical neuron autophagosome shown at higher magnification contains internal membranes, cytoplasm and a mitochondrion. Scale bar, 200 nm. (F) Lower magnification survey of a cortical neuron from a 14-month old *Cln3^Δex1-6^* mouse, containing large amounts of *Cln3*-specific storage material (white arrow). The plasma membrane of the cells and two adjoining cells is indicated (PM). Scale bar, 2 µm. (Nu), nucleus.

Previous analysis of the neuroretina of *Cln3^Δex1^*^-^*^6^* null mutant mice at 12–18 months of age showed numerous membrane-bound structures and autofluorescent storage material in the ganglion and bipolar cells, as well as in the inner segment of photoreceptor cells, which were absent in wild-type mice of similar age, thus mimicking the lysosomal storage of human individuals affected with juvenile NCL (19,20). The greatest amount of autofluorescent storage, the classic marker of NCL disease, was found in the photoreceptor-RPE region (19). Here, as a model to investigate the role of phagocytic mechanisms in this neuropathogenesis and in the early visual deterioration that is a hallmark of human juvenile NCL, we analysed retinal pigment epithelial (RPE) cells of 12–20 month-old wild-type and *Cln3^Δex1^*^-^*^6^* mice at the ultrastructural level using transmission electron microscopy (TEM) (Fig. 1). We found that storage material accumulations consisting of single-membrane bound, multilamellar ‘fingerprint’ type structures, similar to those previously described for juvenile NCL deficient cells, were also present in the RPE of the *Cln3^Δex1^*^-^*^6^* mice. The phenotype was seen across the 12- to 20-month age range and representative examples from a 12-month old *Cln3^Δex1^*^-^*^6^* mouse are shown in Figure 1C. These were not seen in wild-type controls.

**Figure 2.**
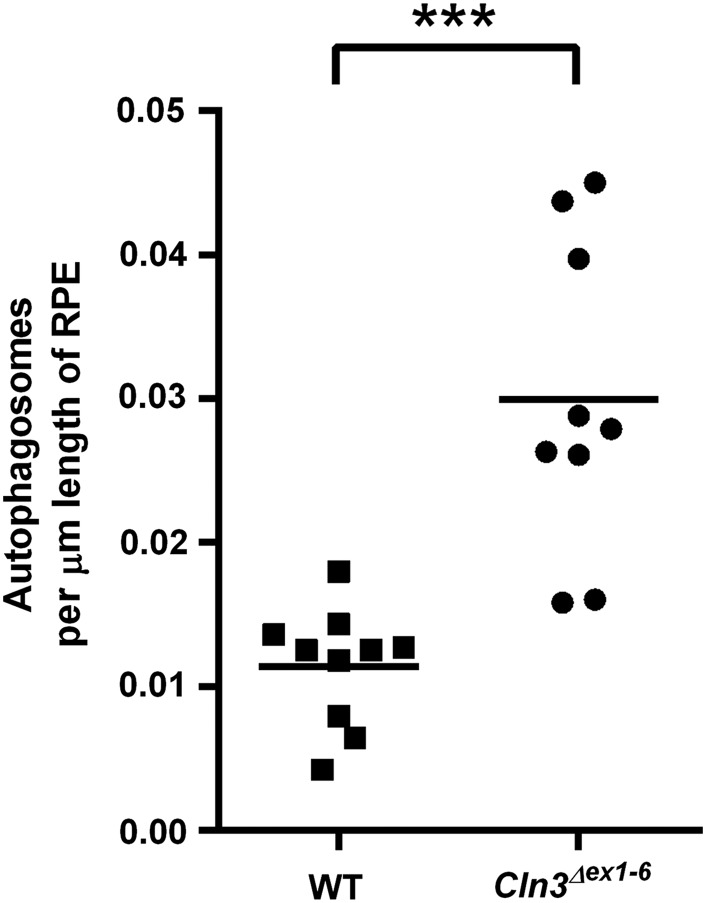
Significant accumulation of autophagic vacuoles occurs in RPE from aged *Cln3^Δex1-6^* mice. Dot plot quantification of the number of autophagosomes per µm of RPE in 12- to 19-month old wild-type (squares) and *Cln3^Δex1-6^* mouse RPE (circles) shows an overall three-fold increase in mutant cells, with the range spanning from an approximately normal number to nearly five-fold increased. RPE was tested in *n* = 10 (wild-type), *n* = 9 (*Cln3^Δex1-6^*) different mice per genotype. Statistical significance measured using the non-parametric Mann and Whitney test, *** indicates *P* < 0.001.

This analysis also revealed a markedly high number of structures with the appearance of autophagosomes, which were easily identified by their internal content containing cytoplasmic structures and membranes, and their characteristic double limiting membrane structure. Representative examples from a 12-month old *Cln3^Δex1^*^-^*^6^* mouse are shown in Figure 1A and B. Their ultrastructures were largely representative of the later maturation stages of autophagy (termed intermediate/degradative autophagic vacuoles, AVi/d), i.e. post-lysosome fusion, acidic and containing lysosomal membrane proteins and enzymes. Ultrastructurally, the mature AVi/d vesicles are defined as having partially degraded contents with some still identifiable cellular material such as rough ER, mitochondria, vesicles (61). Using these criteria to define the autophagosomal structures, their numbers were quantified per length of RPE in both wild-type and *Cln3^Δex1^*^-^*^6^* mice at 12–20 months of age (Fig. 2). Although autophagosomes can be found in RPE of wild-type mice, the *Cln3^Δex1^*^-^*^6^* mice showed on average a three-fold increase in the number of autophagosomes per length of RPE (*P* < 0.0001). These were concentrated in the basal area of the cell towards Bruch's membrane (BrM) in the region where the endo-lysosomal cell compartments are located, and were not seen in the apical region of the RPE cells where their apical processes (ApP) engulf the photoreceptors (Fig. 1A). To our knowledge, this is the first evidence that loss of *Cln3* function gives rise to defects in autophagy in the retina.

Similar progressive accumulation of storage material characteristic of juvenile NCL is well documented in neurons of the brain of *Cln3^Δex1^*^-^*^6^* (55,57) and other *Cln3*-deficient mouse models (60,62). However, despite evidence in a cultured *Cln3*-deficient neuronal precursor cell model for accompanying defects in autophagy (36), only limited *in vivo* assessment for this second finding of autophagy has been performed in the CNS of any *Cln3* mice (55) or human patients with juvenile NCL (56). Using TEM analysis of the cortex of 12- to 14-month old *Cln3^Δex1^*^-^*^6^* mice, we observed neurons with significant electron dense lysosomal storage material, defined by a single limiting membrane, even prior to any signs of significant degeneration of the cell (Fig. 1F), which was of classic ‘fingerprint’ ultrastructure (Fig. 1D). These storage bodies were present alongside double-membrane limited autophagosomes of variable morphology (Fig. 1D and E) that contained multiple internal membranes, sequestered cytoplasm and cytoplasmic contents at different stages of degradation, for example one with a mitochondrion clearly visible (Fig. 1E).

Therefore, the CNS and neuroretina of *Cln3^Δex1^*^-^*^6^* mice similarly display defective lysosomes and autophagolysosome structures, both indicative of deficient lysosome-related degradative processes. This ultrastructural survey suggests that similar lysosome-related degradative defects occur in the RPE and the CNS neurons of *Cln3^Δex1^*^-^*^6^* mice, both highly metabolically and catalytically active cell types. Accumulation of storage material presumably arises from lysosomal deficiency and a consequent reduced autophagic turnover, thereby giving rise to increased autophagosome accumulation (53,63).

### Abnormal storage accumulations in aged *Cln3^Δex1^*^-^*^6^* null mutant RPE are characteristic of juvenile NCL, containing mitochondrial F_0_-ATP synthase subunit c

Recent studies suggest that interactions between the phagocytosis and autophagy pathways within the RPE are essential to vision, through their role in maintenance and turnover of photoreceptors by degradation of their outer segments (54). In view of the juvenile NCL-type ultrastructural defects uncovered in the RPE, we proceeded to further analyse these pathways in *Cln3^Δex1^*^-^*^6^* mice. First, using cryo-immuno EM we investigated wild-type and *Cln3^Δex1^*^-^*^6^* mutant RPE for the presence of mitochondrial F_0_-ATP synthase subunit c protein, the aberrant lysosomal accumulation of which is the hallmark of juvenile NCL in both human and mouse neurons. We used differential immunogold labelling of F_0_-ATP synthase subunit c (smaller, 10 nm gold particles) and cathepsin D (larger, 15 nm gold particles), a label of the lysosomes.

**Figure 3.**
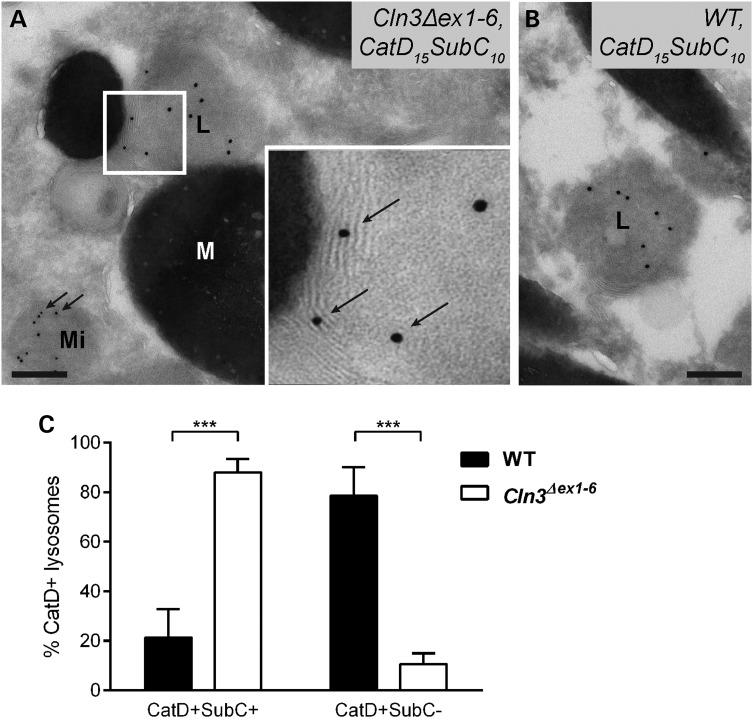
Significant accumulation of cathepsin D and mitochondrial F-ATP synthase subunit c-containing autophagolysosomal structures in RPE of aged *Cln3^Δex1-6^* mice. Cryosections of *Cln3^Δex1-6^* (**A**) and wild-type (**B**) RPE were double-labelled for cathepsin D (large particles, 15 nm gold) and subunit c of mitochondrial ATP synthase (small particles, 10 nm gold). *Cln3^Δex1-6^* RPE shows a four-fold increase in the percentage of cathepsin D positive lysosomes that also stain for subunit c of mitochondrial ATP synthase (indicated by 10 nm gold particles shown with arrows in higher magnification box inset (A) These structures also have nascent fingerprint-like lamellar membrane structures indicative of autophagolysosomal origin in the *Cln3^Δex1-6^* RPE, that are not present in wild-type (visible in the panel A inset, associated with the 10 nm arrowed particles—white boxes). Subunit c of mitochondrial ATP synthase is also present as expected in mitochondria (arrows, main panel of A). Scale bars, 200 nm. (L) lysosome, (M) melanosome, (Mi) mitochondria. (**C**) Bar graph quantification of cathepsin D and subunit c of mitochondrial ATP synthase labelling in *Cln3^Δex1-6^* (white bars) and wild-type (black bars); approximately 25 lysosomes in three different eyes tested per genotype. Statistical significance measured using the non-parametric Mann and Whitney test, *** indicates *P* < 0.001.

In both wild-type and *Cln3^Δex1^*^-^*^6^* null RPE samples, apart from labelling the mitochondria, the labelling for subunit c of mitochondrial F_0_-ATP synthase also localized to the lamellar membranes within lysosomes (Fig. 3A and B). However, consistent with the ultrastructural findings of storage material, we found an average four-fold increase in the number of these cathepsin D-positive lysosomes (larger gold particles) also containing membranes positive for subunit c mitochondrial F_0_-ATP synthase (smaller gold particles) in *Cln3^Δex1^*^-^*^6^* mutant compared with wild-type (Fig. 3C). Normal cathepsin D-positive lysosomes containing no F_0_-ATP synthase subunit c were correspondingly very infrequent in *Cln3^Δex1^*^-^*^6^* RPE, reduced by a similar order of magnitude compared with wild-type. Therefore we concluded that most lysosomes in *Cln3^Δex1^*^-^*^6^* RPE still contain cathepsin D, but show reduced degradative capacity and an autophagolysosomal character as marked by F_0_-ATP synthase subunit c. In addition internal membranes were evident that were suggestive of incompletely digested lysosomal contents (Fig. 3A, insert), akin to the fingerprint bodies seen in conventional EM (Fig. 1C).

### An abnormal accumulation of basally localized phagosomes occurs in aged *Cln3^Δex1^*^-^*^6^* null mutant RPE

The daily photoreceptor renewal cycle, critical for their survival, involves phagocytosis by RPE cells of the spent, shed POS (49). In the RPE, phagosomes engulf the POS at the apical surface, and then rapidly move from the apical to the basal region of the RPE cell after they are formed. During this microtubule-dependent movement of the phagosomes, they are thought to mature by fusing to progressively acidic endocytic vesicles culminating in fusion with lysosomes, thus allowing their internal contents to be degraded (48,49,64). To determine whether there was a defect in this lysosome-related phagosome processing mechanism in *Cln3^Δex1^*^-^*^6^* RPE, eyes were harvested 2 h after light onset, when most shed rod outer segments (ROS) have been engulfed by the RPE and phagosome maturation and degradation is underway.

**Figure 4.**
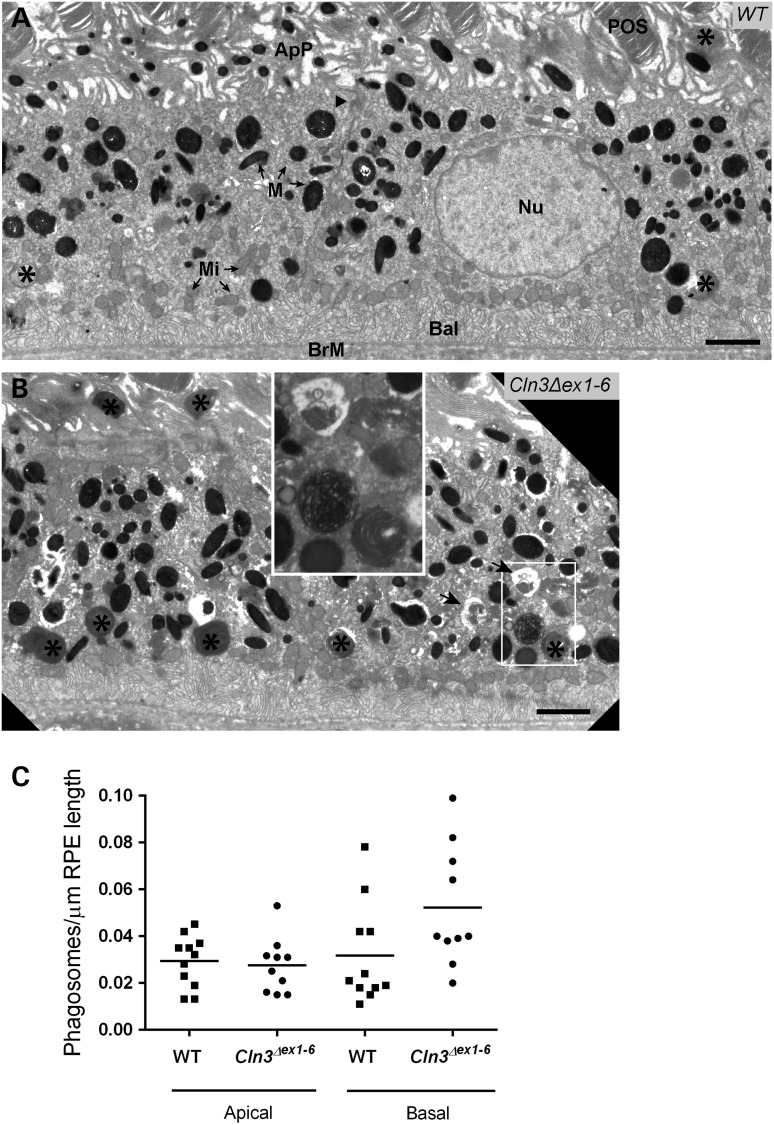
Phagosomes accumulate in the basal region of RPE cells in aged *Cln3^Δex1-6^* mice. Conventional EM analysis of wild-type (**A**) and *Cln3^Δex1-6^* (**B**) RPE shows increased numbers of phagosomes (asterisks), identifiable by their content of membraneous photoreceptor discs, accumulating in the basal region of the cell in *Cln3^Δex1-6^*. Autophagosomes (large black arrows in B) are also observed towards the basal area of the cell in *Cln3^Δex1-6^*, and these are not seen in wild-type cells. The distinction between these two structures is shown at higher power magnification in the inset in B (white boxes). (**C**) Dot plot quantification of the number of apical and basal phagosomes per µm of RPE length in wild-type (squares) and *Cln3^Δex1-6^* RPE (circles). *N* = 11 (wild-type), *n* = 10 (*Cln3^Δex1-6^*) different mice tested per genotype. The difference in basal phagosome numbers does not reach significance. POS, photoreceptor outer segments; Nu, nucleus; ApP, apical processes; BrM, Bruch's membrane; BaI, basal infoldings, Mi, mitochondria (examples arrowed), M, melanosomes (examples arrowed), black arrowhead indicates tight junction. Scale bars, 2 mm.

TEM was used to identify phagosomes, which were easily distinguished by the presence of stacked ROS discs within the phagosome lumen (Fig. 4). We have previously shown that movement of phagosomes from the apical to the basal region of the RPE is co-incident with phagosome maturation (49). In the TEM images we were able to score and classify phagosomes as apical (above the tight junctions) or basal (below the tight junctions) based on their location within the RPE cells (Fig. 4A and B). Whilst the number of apical phagosomes did not change between the *Cln3^Δex1^*^-^*^6^* null mutant and wild-types, there was an increased number of phagosomes in the basal area of *Cln3^Δex1^*^-^*^6^* null RPE cells compared with controls equating to roughly doubled numbers (Fig. 4C). This positional data suggested that loss of *Cln3* function results in defects in a late stage of the phagocytosis process that occurs in the basal region of RPE cells and is associated with the degradation of internalized phagosome contents.

### A delay occurs in the fusion of phagosomes with lysosomes in the RPE of *Cln3^Δex1^*^-^*^6^* null mutants

The internalized POS cargo within phagosomes is degraded during the maturation process that culminates in their fusion with lysosomes to form phagolysosomes. To study the effects on phagosome processing (maturation) of loss of CLN3, we double-labelled retinal sections taken 2 h after light onset. We examined phagolysosome behaviour in more detail using cryo-immuno EM and gold-conjugated antibodies to rhodopsin (15 nm gold particle) and cathepsin D (10 nm gold particle). We have previously shown that following fusion between mature rhodopsin-positive phagosomes and cathepsin D-positive lysosomes, the resulting cathepsin D-positive phagolysosomes initially stain strongly for rhodopsin and the outer segment discs are clearly visible within their lumen (49). Then, as phagosomal degradation proceeds, a reduction in rhodopsin staining is observed, coinciding with loss of visible outer segment discs within the phagolysosome (49).

**Figure 5.**
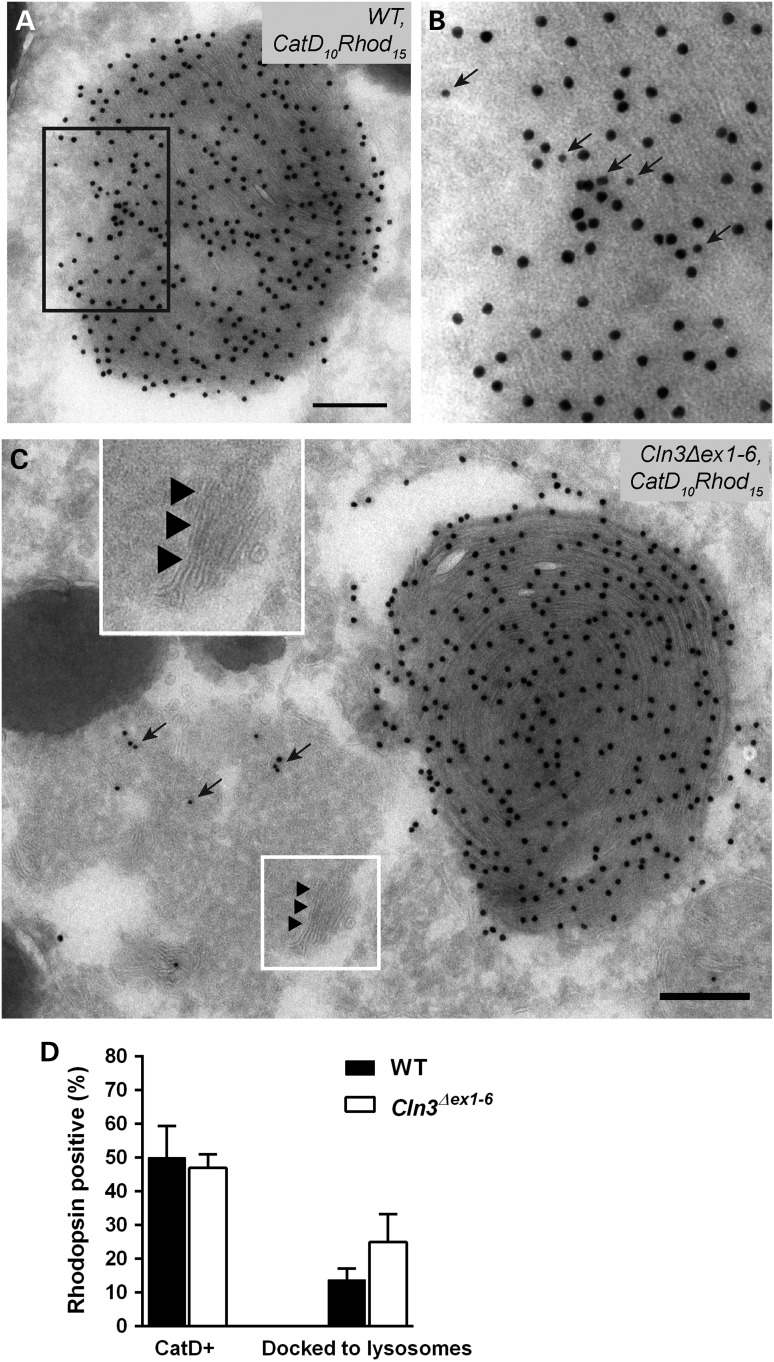
Increase in rhodopsin-containing phagosomes docked to lysosomes without content mixing in RPE cells of aged *Cln3^Δex1-6^* mice. Cryosections of wild-type and *Cln3^Δex1-6^* RPE were double-labelled for rhodopsin (15 nm gold) and cathepsin D (10 nm gold) to distinguish phagolysosomes that are positive for both rhodopsin and cathepsin D from phagosomes that stain only for rhodopsin. A typical phagolysosome from wild-type RPE is shown in (**A**). The boxed area in (A) is enlarged in (**B**). A typical phagolysosome from *Cln3^Δex1-6^* RPE where the lysosomal and phagosomal contents have not mixed is shown in (**C**). Arrows indicate labelling for cathepsin D, arrowheads point at nascent fingerprint-like lamellae exclusively seen in *Cln3^Δex1-6^* RPE cathepsin D-positive structures (also shown in higher magnification inset, C—white boxes). Scale bars, 200 nm. (**D**) Bar graph quantification shows an equivalent percentage of rhodopsin-positive phagolysosome structures that also stain for cathepsin D in *Cln3^Δex1-6^* and wild-type (left-hand bars). The percentage of rhodopsin-positive structures that appear docked to lysosomes but where the phagosomal and lysosomal contents have not mixed is towards two-fold increase in *Cln3^Δex1-6^* compared with wild-type (right-hand bars), but this difference does not reach statistical significance. Labelling of approximately 25 phagosomes was analysed in three eyes per genotype.

In both wild-type and *Cln3^Δex1^*^-^*^6^* null RPE, phagolyosomes could be detected with visible outer segment discs that stained strongly positive for rhodopsin and for cathepsin D, identifying them as phagolysosomes (example shown in Fig. 5A and B). Quantitation of the percentage of rhodopsin-positive structures that also stained for cathepsin D showed no significant change in *Cln3^Δex1^*^-^*^6^* null RPE cells (Fig. 5D). However, in *Cln3^Δex1^*^-^*^6^* null RPE cells there was a higher frequency of profiles where the phagosomal and lysosomal content had not mixed (Fig. 5C and quantitated in D). The lack of content mixing led us to classify these structures as ‘docked’, although frequently no membrane was discernible between the rhodopsin-positive discs and the cathepsin D positive lysosomal content, suggesting a degree of successful fusion. These data imply that there is a defect in a late stage of fusion between phagosomes and lysosomes in *Cln3^Δex1-6^* null RPE cells that could lead to delayed disc degradation and hence the accumulation of basal phagosomes that we identified by conventional TEM (Fig. 4). It is possible that phagosome:lysosome content mixing might need an initial degradative process to break up the lipid-rich POS membranes. Notably, multilamellar ‘fingerprint’ like autophagolysosomal structures typical of juvenile NCL deficient cells, and noted also in Figure 3A insert, could be observed within the lysosomal content of the cathepsin D-positive docked structures in *Cln3^Δex1-6^* (Fig. 5C arrowheads).

### Modelling phagocytic ability by latex bead uptake shows that *Cln3^Δex1-6^* null mutant embryonic fibroblasts display phagosome processing defects

Although POS shedding is regulated by light and circadian rhythm, the subsequent process of phagocytic engulfment, processing and degradation is relatively unsynchronized, making it difficult *in vivo* to dissect the effects of CLN3 loss on the sequential events in phagosome processing. We therefore turned to a more experimentally tractable system for analysis of the phagocytic pathway, using embryonic fibroblasts isolated from *Cln3^Δex1-6^* and wild-type mice (MEFs). These cells can be used to analyse phagocytosis after transfection with phagocytic receptors to obtain ‘engineered phagocytes’ (65,66) that have the capacity to recognize and internalize opsonized particles fed to the cell.

**Figure 6.**
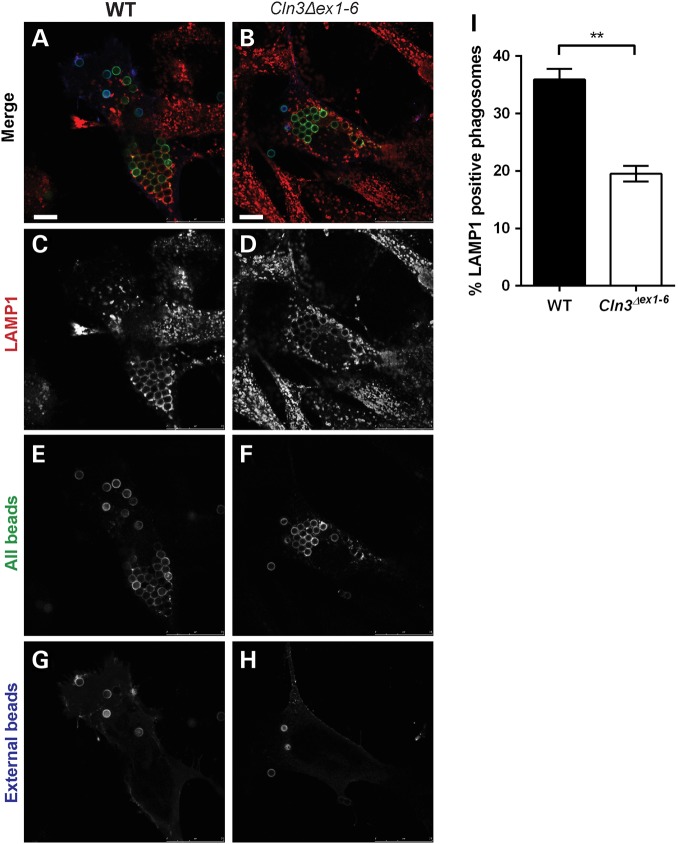
Significant decrease in phagosome maturation in *Cln3^Δex1-6^* MEFs, marked by reduced acquisition of the lysosomal marker LAMP1. Wild-type (**A**, **C**, **E** and **G**) and *Cln3^Δex1-6^* (**B**, **D**, **F** and **H**) MEFs were incubated with latex beads 24 h after transfection with FcγRIIA. Phagosome maturation was allowed to proceed for 60 min before cells were prepared for indirect immunofluorescence studies. LAMP1 (C and D) was detected with an anti-rat secondary antibody conjugated to Alexa594 (red), all beads (E and F) were stained with an anti-IgG secondary antibody conjugated to Alexa488 (green) and external beads (G and H) were stained with an anti-IgG secondary antibody conjugated to Alexa680 (far red). (**I**) Bar graph quantification shows a two-fold decrease in LAMP1-positive phagosomes in *Cln3^Δex1-6^* compared with wild-type cells; 100 phagosomes from ≥20 cells were analysed per genotype. Statistical significance measured using the unpaired Student's *t*-test, ** indicates *P* = 0.002. Scale bar, 10 m.

FcγRIIA-expressing *Cln3^Δex1-6^* and wild-type mouse embryonic fibroblasts (MEFs) were incubated with IgG-opsonized latex beads for 1 h at 37°C to allow for phagosome maturation. Labelling of external beads with far red-conjugated anti-IgG antibody at 4°C allowed the distinction between surface and internalized beads, and this showed that both wild-type and *Cln3^Δex1-6^* MEFs were able to form phagosomes that internalized the opsonized beads (Fig. 6A, B and E–H). Subsequently, cells were fixed and stained with LAMP1 to analyse phagosome-lysosome fusion, since LAMP1 is a marker of late endosomes and lysosomes that is known to be delivered to phagosomes during the maturation process, after the fusion of phagosomes with lysosomes (66). We found that whilst 36% of the internal phagocytosed beads stained positive for LAMP1 in wild-type MEFs, <20% of beads in *Cln3^Δex1-6^* MEFs were LAMP1 positive (Fig. 6C, D and I). Anecdotally it also appeared that the intensity of the LAMP1 staining of induced phagosomes might be reduced in the *Cln3^Δex1-6^* mutant cells, consistent with reduced LAMP1 recruitment to the phagosome membranes.

These results show that although MEFs lacking CLN3 are able to phagocytose materials, the delivery of lysosomal markers to the phagosomes is significantly reduced. In the context of the findings in RPE (Fig. 5), this delay or partial block in phagosomal maturation provides supporting evidence of deficient fusion/content mixing phagosomes and lysosomes in *Cln3*-deficient fibroblasts.

### Phagosomes in *Cln3^Δex1-6^* null mutant embryonic fibroblasts display morphological defects suggestive of impaired processing via lysosome fusion (maturation)

**Figure 7.**
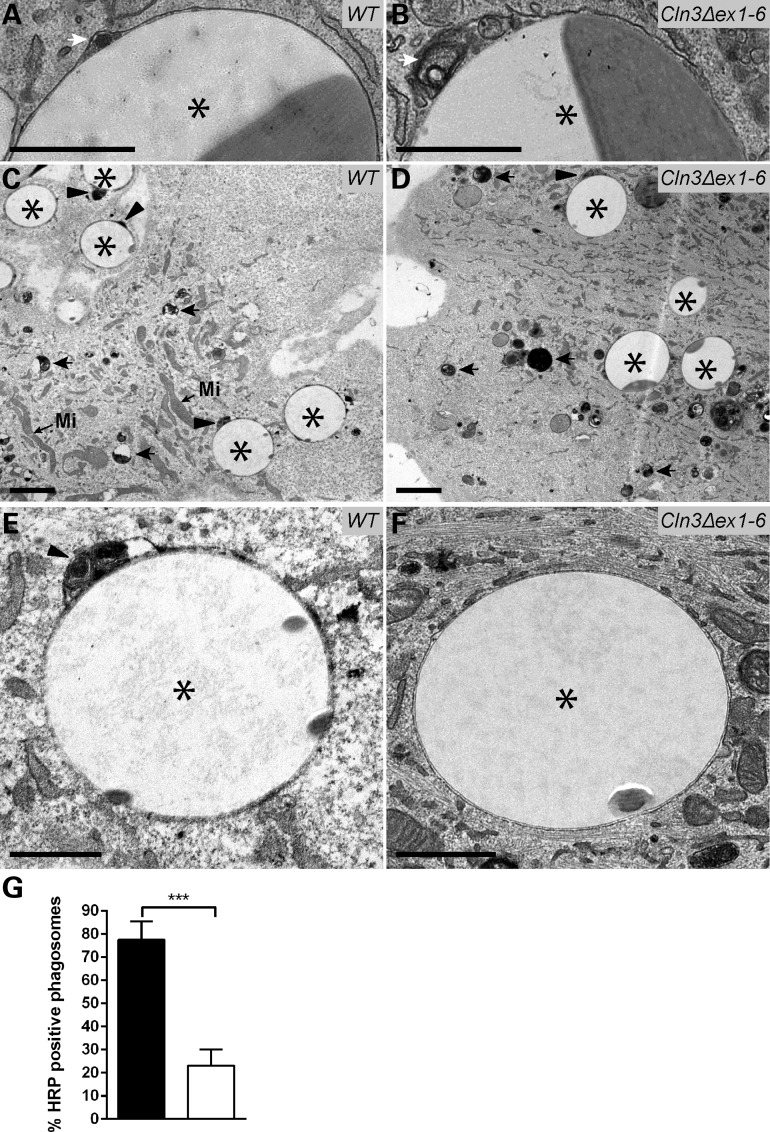
Ultrastructural analysis shows that the fusion of lysosomes with phagosomes is deficient in *Cln3^Δex1-6^* MEFs. (**A** and **B**) Conventional EM analysis of FcγRIIA-transfected wild-type and *Cln3^Δex1-6^* MEFs incubated with latex beads for 60 min to allow for phagosome maturation. In wild-type, there is a smooth, uninterrupted membrane surrounding the phagocytosed latex beads (asterisk, A), with the phagosome lumen visible in between the phagocytic membrane and the bead (white arrow, A). In contrast, phagosomes in *Cln3^Δex1-6^* have a disorganized membrane surrounding the beads (asterisk, B), with membranes and vesicles accumulated between the phagocytic membrane and the bead and visible in the lumen (white arrow, B). (**C–F**) Conventional EM analysis of wild-type and *Cln3^Δex1-6^* MEFs transfected with FcγRIIA receptor, incubated with HRP for 90 min then chased for 5 h to allow for HRP-labelling of lysosomes. After chasing, cells were incubated with latex beads for 60 min to allow for phagosome maturation. Both wild-type and *Cln3^Δex1-6^* contain HRP-labelled lysosomes (back arrows in (C and D)). In wild-type, HRP is also localized in phagosomes marking successful phagosome-lysosome fusion (arrowheads in C and E). In contrast in *Cln3^Δex1-6^*, HRP is rarely observed in phagosomes, and when occasionally present is at much lower electron density (arrowhead in D). Most phagosomes contain no HRP indicating a lack of lysosome fusion events (D and F). Mi, small black arrows indicates mitochondria; black asterisks indicate latex beads/phagosomes. (**G**) Bar graph quantification of 100 HRP-positive phagosomes per genotype shows a four-fold decrease in *Cln3^Δex1-6^* (white bar) FcγRIIA-transfected MEFs compared with wild-type (black bar); 100 phagosomes from ≥20 cells were analysed per genotype. Statistical significance measured using the unpaired Student's *t*-test, *** indicates *P* < 0.001. Scale bars, 1 µm in A, B, E, F and 2 µm in C, D.

We next investigated the effects of CLN3 loss on phagosome-lysosome fusion at the ultrastructural level in *Cln3^Δex1-6^* MEFs. First, a structural analysis was made on the FcγRIIA-transfected *Cln3^Δex1-6^* and wild-type MEFs that had been allowed to internalize opsonized beads for 1 h at 37°C to allow for phagosome maturation, as previously. In wild-type cells, all the latex beads were surrounded by a smooth phagosomal membrane, with the phagosome lumen visible to one side of the phagosome between its membrane and the surface of the latex bead (Fig. 7A). Occasionally a few vesicles were observed within the lumen, suggesting that fusion events with the endo-lysosomal pathway had happened as part of the normal maturation process. The phagosomes in *Cln3^Δex1-6^* cells in contrast, displayed accumulations of membranous and vesicular structures in the lumen and also around the rest of the phagosome, visible between the bead and the phagosomal membrane (Fig. 7B). This suggested that similar endo-lysosomal fusion events have occurred, presumably involving early endosomes and multivesicular bodies, but that phagosomal maturation is delayed or partially arrested.

Next, FcγRIIA-transfected *Cln3^Δex1-6^* and wild-type MEFs were incubated for 90 min with the endocytosis fluid-phase marker horseradish peroxidase (HRP) then chased for 5 h with medium containing no HRP. Under these conditions, HRP is cleared from endosomes and accumulates in lysosomes where it is resistant to lysosomal degradation (67). The cells were then exposed to opsonized latex beads for 1 h to allow for phagosome maturation, before being processed for TEM analysis. HRP-loaded lysosomes were clearly visible within both the wild-type and mutant cells (Fig. 7C and D). In the great majority of phagosomes in the wild-type cells, there was HRP labelling that demonstrated delivery of electron-dense material characteristic of lysosomes to the phagosome lumen (arrowheads in Fig. 7C and E). This indicated successful fusion of HRP-loaded lysosomes with latex bead-containing phagosomes, similar to the images captured previously by others using this technique (66). In contrast, in the *Cln3^Δex1-6^* MEFs, HRP labelling was weak if present at all, and largely absent within phagosomes. The phagosomal membrane was again clearly less smooth and contained internal membranous vesicular structures (Fig. 7D and F). This suggests that vesicle fusion can occur in CLN3-deficient cells, but that phagosomal maturation is delayed or arrested.

These findings indicate that HRP is able to reach the lysosomes of *Cln3^Δex1-6^* MEFs, where phagosomes also can form, but that HRP-tracked lysosome fusion with phagosomes is drastically reduced showing that their maturation and processing is deficient. Quantitative analysis showed a three-fold reduction in the percentage of latex bead-containing phagosomes that were positive for HRP in *Cln3^Δex1-6^* MEFs (Fig. 7G). This strikingly different phenotype concurs with that observed in *Cln3^Δex1-6^* RPE cells, where the contents of lysosomes often failed to mix with that of the phagosomes (Fig. 5C and D).

## Discussion

Four *Cln3*-deficient mouse models of juvenile NCL (57,59,60,68) display significant and progressive disease-specific lysosomal storage accumulation. However they have clinically mild retinal degeneration with minimal retinal cell loss (16,19,20,55,62,69), unless present on a mixed CD1 albino background (60). This mouse-human difference may be influenced by differences in numbers and distribution of photoreceptors, life spans and/or disease progression rates (in particular for slower degenerating conditions such as juvenile NCL), as seen for other mouse models of neuroretinal disorders (70). Here, by ultrastructural examination of the RPE we detail the retinal degeneration that occurs in aged *Cln3^Δex1-6^* mice. This model allows a first analysis of what is probably the earliest cell biology of CLN3 eye disease, and of previously uncharacterized disturbances underlying this devastating condition. Here, we describe cell biological disruption affecting two distinct lysosome-mediated degradative pathways within the RPE: autophagy and phagocytosis.

We previously described an increase in autofluorescent material in the *Cln3^Δex1-6^* photoreceptor-RPE layer (19). Here, we detect significant abnormalities affecting the lysosomes of the RPE cells, showing storage of mitochondrial F_0_-ATP synthase subunit c with the juvenile NCL-characteristic ‘fingerprint’ ultrastructural appearance in the majority of them, akin to the lysosomal storage material detected in the CNS and retina of affected individuals (10,11). This is supported by cryo-immuno EM showing a majority of cathepsin D-positive lysosomes with these autophagolysosomal characteristics, and a corresponding reduction in normal lysosomes. In the RPE the fingerprint-like inclusions and subunit c staining are not as prominent as found in the brain even of younger CLN3-deficient mouse models, indeed they were initially missed even at late ages (19), nor have storage bodies been reported in the RPE of any CLN3-deficient mice before. It is possible this pathology might become more prominent in older mice, or this might reflect a real difference in the ability to degrade cellular material in the RPE compared with neurons. The relative lack of visual impairment in all CLN3-deficient mice contrasts with human juvenile NCL where degenerating vision is the lead symptom, and progression to blindness tends to be rapid, although disease does not tend to become apparent until later childhood around 5–10 years of age. Affected individuals can also have an extended period, up to several years, with only visual symptoms before any evidence of CNS involvement and a diagnosis of NCL (18). The early retinal disease in human juvenile NCL has never been studied for comparison, since publications on retina of affected individuals are all post-mortem, at a point where significant retinal degeneration has already occurred.

Alongside visible changes to the lysosome compartments, there is an accumulation of late-stage autophagosomes (autophagolysosomes), as well as late-stage (basal) phagosomes (phagolysosomes) in *Cln3^Δex1-6^* RPE. We suggest that these events arise as a consequence of defective degradative capacity of the lysosomes, which interferes with the processing steps of both these pathways that require interaction with the endo-lysosomal system and culminate in fusion with the lysosomes for delivery of their degradative contents. The cryo-immuno EM analysis offers support for a specific post lysosome fusion defect, at least in phagocytosis, that prevents efficient delivery and mixing of lysosomal contents to achieve this endpoint. Content mixing of two distinct compartments—the lysosome and the phagosome, with its lipid-rich/insoluble contents—could require an initial degradative process to break up the POS lamellae.

Analysis of liver extracts from *Cln3^Δex7/8^* mice has already implicated defects in autophagic maturation in the pathogenesis of juvenile NCL disease, with accumulations of mature, post-lysosomal fusion stage AVi/d-type autophagosomes (36). In the *Cln3^Δex1-6^* RPE the three-fold increase in late autophagosomes suggests they form and can fuse with lysosomes, but are failing in the final steps of the autophagy maturation/degradation process for digestion of internal contents. Potentially, their production is also over-stimulated in the CLN3-deficient RPE. It was previously proposed that impaired autophagosome:lysosome fusion is a global problem in LSDs (6,8) with build-up of autophagic substrates such as aggregate-prone proteins and dysfunctional mitochondria; a lack of nutrient recycling could potentially trigger further autophagy in an attempt by the cell to compensate.

Cryo-immuno EM confirms that in both *Cln3^Δex1-6^* and control RPE, phagolysosomes containing mixed cathepsin D and rhodopsin-positive POS are present, i.e. that lysosomal fusion and hydrolase delivery to phagosomes can occur in mutants, but we found almost doubled numbers of phagosomes compared with wild-type accumulated in the basal RPE region where lysosomal fusions take place. In phagocytosis, although the molecular regulation of phagosome engulfment by the RPE is well studied, less is known about the subsequent phagosome processing that involves movement of the phagosomes from the apical site of engulfment to the basal region for lysosomal fusion (64,71,72). By analogy with other professional phagocytes this is likely to involve sequential interactions with the endocytic pathway in order for the phagosome to acquire the capacity to fuse with the lysosome (73,74). Consistently, we recently showed that sequential stages in phagosome maturation in RPE cells were co-incident with interaction with the endocytic pathway (49). Furthermore phagosome maturation was co-incident with movement of the phagosome from the apical to the basal region of the cell.

As the number of apical phagosomes and the length of POS do not vary between *Cln3^Δex1-6^* and wild-type RPE, the shedding and phagocytic uptake of shed POS does not seem affected. The accumulation of basal but not apical phagosomes in the RPE 2 h after light onset, such that early stages of phagosome maturation appear relatively unaffected, contrasts with the *shaker-1* mouse model of Usher syndrome 1B. This carries a mutation in myosin VIIa and apical phagosomes accumulate, indicating a role for myosin VIIa in phagosome movement and/or maturation (75).

In *Cln3^Δex1-6^*, the accumulation of docked or partially fused phagosomes with lysosomes suggests a role for CLN3 at a late stage in phagosome processing/degradation. Akin to autophagy, this could reflect some level of deficient lysosome fusion and/or an inability to degrade internal contents once fused to lysosomes. Since we found that approximately twice as many phagosomes were docked to lysosomes compared with wild-type, many showing little or no mixing of cathepsin D-positive lysosomal and rhodopsin-positive phagosomal content, it seems likely that phagosome-lysosome fusion in *Cln3^Δex1-6^* RPE may occur at a slower pace than in wild-type.

Delayed phagosomal processing was further confirmed in a model phagocytic system, MEFs expressing FcγRIIA allowed to phagocytose opsonized latex beads. This system showed reduced fusion of phagosomes with LAMP1 positive or HRP-preloaded lysosomes, with abnormalities of the phagosome membranes and the processing of their internal contents. These findings support the suggestion that fusion and processing are both delayed or partially blocked, and suggests a general, rather than an RPE specific, role for CLN3 in phagosome degradation.

The accumulation of lipofuscin-containing deposits, believed in part to be derived from the products of phagocytosis, frequently correlates with retinal degeneration in mouse models of age-related macular degeneration, and inherited retinal degenerations (76). For example, the *mcd/mcd* mouse model that expresses an inactive form of cathepsin D (the CLN10 gene causing congenital NCL), leading to a major block in phagosome degradation in the RPE, exhibits a severe retinal degeneration (77). The more subtle inhibition of phagosome degradation in the RPE of the *Cln3^Δex1-6^* mouse model does not lead to the retinal degeneration found in human patients, although accumulation of autofluorescent material and apoptosis are present in the neural retina (19). Interestingly, the *shaker-1* mouse recapitulates the hearing and vestibular defects of Usher syndrome 1B, but does not show the retinal degeneration characteristic of human patients (75). Taken together these data suggest that defects in phagosome degradation in the mouse RPE may have more subtle effects on the health of the neural retina than in human patients.

Another inherited retinal degeneration characterized by partial defects in phagosome degradation in the RPE is x-linked choroideremia, caused by loss of function of Rab Escort Protein 1 (Rep1) leading to defective Rab prenylation. Using tissue restricted mouse models we have shown that delayed phagosome degradation caused by loss of Rep1 in the RPE does not on its own lead to significant photoreceptor degeneration but exacerbates the photoreceptor degeneration caused by loss of Rep1 in photoreceptors (78,79). This would be consistent with the possibility that delayed phagosome degradation due to loss of Rep1 or CLN3 may not alone cause retinal degeneration, but could compromise RPE function sufficiently to increase the susceptibility of the neural retina to cell autonomous defects.

In conclusion, the degradative compartments of the autophagy and phagocytosis pathways that are key to phagocytic POS turnover by the RPE can form in *Cln3^Δex1-6^* RPE, but we find a significant disruption to the terminal-stage processing which ultimately relies on the lysosome fusion-mediated delivery of hydrolytic enzymes to degrade and recycle the autophagosome and phagosome contents. It has been shown that LSDs may cause cell degeneration and death potentially as a result of the involvement of lysosomes in several vital processes (8). CLN3 is reported to have a number of roles in potentially multiple lysosome-directed cellular pathways that affect intracellular membrane dynamics and protein trafficking. Of particular note for this study are the suspected roles for CLN3 in positioning and motility of late endosomal/lysosomal compartments and its interactions with Rab GTPases (guanosine triphosphate-binding enzymes), and also with Hook 1 which regulates endosome maturation (80,81). Loss of CLN3 has been shown to cause the accumulation of mannose 6 phosphate receptor in the TGN and, thus, reduce delivery of newly synthesized hydrolytic enzymes to the lysosome (28). A reduced concentration of lysosomal enzymes could contribute to the reduction in efficiency of phagosome degradation. The high phagocytic load of the RPE could render these cells particularly sensitive to the effects of compromised lysosomal function. Additionally, CLN3 could modulate the traffic of regulators of phagosome-lysosome fusion or phagosome/lysosome acidification.

This is to our knowledge the first demonstration of a defect in the RPE caused by loss of CLN3. We propose that defects in phagosome degradation may contribute to the retinal degeneration that is an early hallmark of juvenile NCL. Phagosomal defects represent a previously uncharacterized biomarker of early disease, with potential to be a useful assay for monitoring the retinal aspects of disease progression in models of CLN3 deficiency. This could assist in evaluation of future therapeutic approaches to restore vision in individuals affected by juvenile NCL.

## Materials and Methods

### Mice

Knockout mice were made by using an insertion mutation to disrupt the *Cln3* gene, whereby *Cln3* exon 1–6 including the start methionine is replaced by a neomycin selectable marker cassette to create a null allele (57,58). *Cln3^Δex1-6^* homozygotes were maintained on a congenic C57BL/6J background and compared with age-matched wild-type strain for experiments. All animals were kept in accordance with the Animals (Scientific Procedures) Act 1986 of the UK Government, and with the Medical Research Council guidance in ‘Responsibility in the Use of Animals for Medical Research’ (July 1993). Eyes and brains were dissected from aged mice at 12–20 months postnatally, using three ages that showed similar data: 12–13 months (*N*= 5), 16–17 months (*N* = 3) and 19–20 months (*N* = 3).

### Preparation of primary mouse embryonic fibroblasts

Embryos were collected at 13–14 days gestation, the carcasses removed in PBS and the remaining material was minced then placed into 0.25% trypsin (Life Technologies Ltd. 15050-065) in Dulbecco's Modified Eagle Media with GlutaMAX (Life Technologies Ltd. 61965-026) then incubated at 37°C for 20 min with pipette-trituration. The supernatant was removed into an equal volume of media and the MEFs were pelleted then plated out at a density of 10^6^ cells per 9 cm culture dish. MEFs were maintained at 37°C in Dulbecco's Modified Eagle Media with GlutaMAX supplemented with 10% fetal calf serum (Sigma F9665) and 10 µg/ml of gentamicin (Life Technologies Ltd. 15750-037).

### Conventional transmission electron microscopy

Mouse eyes were fixed in 2% paraformaldehyde and 2% glutaraldehyde in 0.1 M cacodylate buffer. MEFs on coverslips, or whole mouse brains were fixed in 2% paraformaldehyde and 1.5% glutaraldehyde in 0.1 M sodium cacodylate buffer. For fixed eyes, the cornea was cut off and the lens removed before postfixing. The eyes were postfixed in 1.5% potassium ferricyanide and 1% osmium tetroxide for 2 h on ice, the MEFs in 1% osmium tetroxide and 1.5% potassium ferrocyanide for 1 h at 4°C, and the brains similarly postfixed but in 1% osmium tetroxide in 0.1 M cacodylate buffer. The MEFs were subsequently incubated in 1% tannic acid in 0.05 M sodium cacodylate. Postfixed material was then dehydrated in ethanol (70%, 90% and absolute) and propylene oxide and transferred to 1:1 propylene oxide:Epon overnight (or for MEFs to a mixture of 1:1 propylene oxide and Epon 812 for 1 h), followed by two changes and embedding in Epon 812. Ultra-thin sections were stained with lead citrate before examination. Eyecups were cut parallel and adjacent to the optic nerve in order to visualize peripheral and central retina. Samples were viewed on a JEOL 1010 (eyes, brains) or G2 Spirit (MEFs) transmission electron microscope. Images were taken with a Gatan Orius SC100B charge-coupled device camera and analysed with Gatan Digital Micrograph (eyes, brains) or acquired with a SIS Morada CCD camera (MEFs).

### Cryo-immuno electron microscopy

Eyes were fixed in 4% paraformaldehyde and 0.1% glutaraldehyde in 0.1 M phosphate buffer. The cornea was cut off to remove the lens. After cutting off the cornea to remove the lens, the retina was cut in small pieces, embedded in gelatine and infused with 2.3 M sucrose. 70-nm sections were cut at −120°C and picked up in 1:1 2% methylcellulose: 2.3 M sucrose. Sequential double labelling with different sizes of gold particles was done as previously described in (82). Phagosomes and phagolysosomes were labelled with anti-rhodopsin RetP1 (Abcam, UK) followed by a rabbit-anti-mouse bridging antibody (Dako, UK) and anti-cathepsin D (Millipore, UK), respectively. Subunit c of the mitochondrial F_0_-ATP synthase was labelled with rabbit antibody (kind gift from Eiki Kominami, Juntendo University, Japan). All antibodies were detected with protein A gold (PAG) (CMC, University Medical Center Utrecht). Samples were viewed on a JEOL 1010 transmission electron microscope and images taken with a Gatan Orius SC100B charge-coupled device camera and analysed with Gatan Digital Micrograph, Adobe Photoshop and ImageJ softwares.

### Quantification of autophagosomes and phagosomes in RPE

The different degradative compartments investigated are as follows: mature autophagosomes and basal phagolysosomes are the terminal degradative compartments of the autophagy and phagocytosis pathways, respectively. Autophagolysosomes and phagolysosomes are terms used to describe the compartments within these two pathways that have undergone lysosome fusion which facilitates breakdown of internal components. To quantify the number of phagosomes and autophagosomes in conventional TEM, an average 1 mm of RPE length was analysed and the position of the phagosomes was recorded as apical if above the tight junction. All phagosomes below the tight junction were scored as basal. All autophagosomes were counted together in all positions in the cells. For quantification of phagolysosomes in cryo-immuno EM, the labelling of approximately 25 phagosomes was analysed in three eyes. Phagosomes positive for rhodopsin only were scored as phagosomes and phagosomes positive for both rhodopsin and cathepsin D were scored as phagolysosomes. Controls for non-specific binding that excluded the primary antibody established that organelles must have a minimum of three gold particles to be classified as positive. To measure mitochondrial F_0_-ATP synthase subunit c labelling in lysosomes we analysed approximately 25 lysosomes in three eyes. Lysosomes were identified by their labelling for cathepsin D. To determine the significance of the data, a *P*-value under 0.05 was considered statistically significant.

### Cell culture and phagocytosis assays

MEFs were transfected with cDNAs encoding human Fcγ receptor FcγRIIA (65) using an Amaxa nucleofector (Lonza AG) and a protocol adapted from (66). At 24 h post-transfection, cells were incubated at 37°C in serum free DMEM media (Sigma) for 1 h (to allow phagosome maturation/lysosome fusion) with 3 um latex beads (LB30 Sigma) that had previously been opsonized with 1 mg/ml human IgG (Sigma) (overnight rotating on ice in human serum). Phagocytosis was synchronized by spinning the cells down at 300 g for 1 min after addition of beads. Cells were then triple stained on ice. They were stained with Alexa 680-conjugated (far red) secondary antibody against human anti IgG (Life Technologies Ltd.) to stain all the external beads, then 4% PFA fixed and stained with Alexa 488-conjugated (green) secondary antibody against human IgG (Life Technologies Ltd.) to label all the beads and an Alexa 594-conjugated (red) secondary antibody against LAMP1 (Abcam ab19294). Samples were imaged using a Leica SPE confocal microscope and images were analysed with Adobe Photoshop. To analyse fusion of phagocytosed latex beads with the lysosomes, cells transfected with FcγRIIA receptor 24 h earlier were fed with 4 mg/ml HRP (Sigma) for 90 min, then chased for 5 h with fresh medium and subsequently fed with 3 µm opsonized latex beads for 1 h. The HRP was revealed by addition of diaminobenzidine and hydrogen peroxide; cells were then processed for TEM analysis.

## Funding

This research was supported by the UK Batten Disease Family Association; by a European Commission 6th Framework Research Grant (LSHM-CT-2003-503051 awarded to H.M.M.); by the Medical Research Council UK (Grant P13251 awarded to M.C.S. and C.E.F.); and the Wellcome Trust (Grant 093445 awarded to M.C.S. and C.E.F.). The Medical Research Council UK funded work at the Laboratory for Molecular Cell Biology (D.F.C). Funding to pay the Open Access publication charges for this article was provided by UCL.
